# Neonatal characteristics and neurodevelopmental phenotypes in congenital cytomegalovirus

**DOI:** 10.1038/s41390-025-04327-z

**Published:** 2025-09-09

**Authors:** Sarah A. Pollick, Fatima Noorulla, Gail Demmler Harrison, Samantha Nikirk, Jenna Hijawi, Julie Sturza, Megan H. Pesch

**Affiliations:** 1 Division of Developmental and Behavioral Pediatrics, Department of Pediatrics, University of Michigan Medical School, Ann Arbor, MI USA; 2 Division of Pediatric Infectious Diseases, Department of Pediatrics, Baylor College of Medicine, Houston, TX USA; 3 Division of Gastroenterology & Hepatology, Department of Internal Medicine, University of Michigan Medical School, Ann Arbor, MI USA; 4 University of Michigan Medical School, Ann Arbor, MI USA; 5 Department of Pediatrics, University of Michigan Medical School, Ann Arbor, MI USA

## Abstract

**Background:**

Children with congenital cytomegalovirus (cCMV) have a wide spectrum of possible neurodevelopmental outcomes.

**Objectives:**

To describe neurodevelopmental (ND) Phenotypes of children with cCMV based on medical, developmental, and behavioral outcomes in childhood, and examine whether birth characteristics were associated with ND Phenotype.

**Methods:**

Caregivers of children with cCMV (*N* = 242, child aged 12 months to <11 years) completed survey instruments reporting on the child’s birth characteristics, reasons for cCMV testing, and present medical, developmental, and behavioral status. Latent Class Analysis was used to derive empirically driven groupings of outcomes, which we called ND Phenotypes. Chi-square analyses and *t*-tests examined the association between ND Phenotypes with child characteristics.

**Results:**

Five ND Phenotypes were identified: 1) No sequelae with or without Isolated SNHL (*n* = 44, 18%), 2) High Medical and Neurodevelopmental Involvement (*n* = 31, 13%) 3) Some Developmental Delays with Mild Neuromuscular Involvement (*n* = 49, 20%) 4) Isolated Speech and Language Delays (*n* = 89, 37%) and 5) Autism and Global Developmental Delay (*n* = 29, 12%). ND Phenotype membership was predicted by birth characteristics and reason for cCMV testing.

**Conclusion:**

Five ND Phenotypes of children with cCMV were identified. Future studies should examine stability of class membership over time.

**Impact:**

- Five neurodevelopmental phenotypes of children with congenital cytomegalovirus were identified.
- All but one phenotype consisted of children with multiple long-term medical and neurodevelopmental sequelae.
- Physical findings, prematurity and intensive care admission at birth were associated with severity of outcome.
- Children tested for congenital cytomegalovirus due to a failed newborn hearing screen alone were most likely to have no long-term developmental delays.

## Background

Congenital cytomegalovirus infection (cCMV) is the most common congenital infection in the United States.^1^ Congenital CMV can result in a wide spectrum of possible neurodevelopmental sequelae.^2,3^ These sequelae range from no appreciable impacts to medically complex children with multiple disabilities. While most children born with cCMV *infection* (otherwise known as asymptomatic or inapparently infected) will have typical neurodevelopmental outcomes, 15% will develop sensorineural hearing loss (SNHL), and up to 50% will have vestibular disorders.^4–6^ Of the roughly 10% born with cCMV *disease*, meaning those with clinically appreciable signs at birth (also known as symptomatic), most children with cCMV disease develop disabilities such as SNHL, vestibular dysfunction, vision loss, epilepsy, and/or cerebral palsy.^7–9^ Yet, there are few nuanced predictors of long-term neurodevelopmental outcomes based on birth features.^10,11^ As such, it is challenging for clinicians to provide reliable tailored anticipatory guidance or recommendations for early intervention. Unsurprisingly, families of infants and children with cCMV report feeling elevated levels of uncertainty and anxiety for their child’s future.^12–14^

Studies examining predictors of cCMV outcomes have largely focused on hearing status, finding associations between cCMV disease (vs. cCMV infection) and first trimester infection with cochleovestibular dysfunction in childhood.^10,11,15^ While understanding a child’s likelihood of developing SNHL is important, it may only be one piece of a more complicated neurodevelopmental outcome. Infants with symptomatic cCMV disease frequently experience several conditions alongside SNHL, including autism, cerebral palsy, or intellectual disability, which can significantly impact their long-term outcomes.

Prior studies have examined specific outcomes (e.g., autism alone, or SNHL alone)^16–19^ or have examined developmental delays without considering the child’s underlying medical conditions.^2^ These studies overlook key context that are critical to understanding a child’s developmental trajectory and functional outcomes.^20^ For instance, knowing a child’s risk of SNHL is only part of the picture – it’s also important to consider the risk of concomitant disabilities such as vision loss, cerebral palsy or intellectual disability.

A better understanding of ND outcomes in children with cCMV, including patterns in overlapping sequelae and predictors of those outcomes is important for tailored anticipatory guidance for families of young children. Therefore, the objectives of this study were 1) to describe ND Phenotypes of children with cCMV using a child-centered approach based on medical, developmental, and behavioral outcomes in childhood, and 2) to examine whether birth characteristics, reasons for cCMV testing and sociodemographic characteristics were associated with ND Phenotype.

## Methods

### Participants and recruitment

Caregivers of children with cCMV were invited to participate in a study examining “the development and behavior” of children with cCMV. Participants were recruited via targeted social media posts to cCMV parent groups, cCMV non-profit newsletter advertising and flyers posted at cCMV-related family events between June 2023 to March 2024. These flyers and social media posts consisted on an image of a mother and a child a gross motor disability, and read “Do you have a child with congenital CMV? Researchers at the University of Michigan want to learn more about their development and behavior”. Participants could scan a QR code or click a link which took them directly to a screener survey. Several participants re-posted the advertisement through their own personal social media profiles. Eligibility requirements were being: 1) the parent or caregiver of a child currently aged 12 months to <11 years with cCMV, including asymptomatic *infection* as well as symptomatic *disease* 2) able to comfortably read and answer questions in English, and 3) over the age of 18 years. Congenital CMV status was reported by caregiver as being either a) confirmed (verified by testing of the amniotic fluid, saliva, urine or blood for the presence of the virus collected before 21 days of age) or b) suspected (antibody testing only performed or testing for the presence of the virus completed on a sample after 21 days of age only). A total of 259 individuals completed the screener portion of the survey, and 243 were eligible. Of those, 242 caregivers completed the questionnaires included in this analysis, one participant did not complete any questionnaires. Those who were found to be ineligible to participate in the survey were due to child being outside the eligible age range (*N* = 15), and child not having confirmed cCMV (*N* = 2).

Participants provided consent to participate and did not receive monetary compensation for completion of the study but were sent a card and a CMV awareness decal as a token of appreciation from the study team. This study was deemed exempt by the University of Michigan Institutional Review Board (HUM00171990).

### Study measures

For the parent study, participants were invited to complete four questionnaires using a link to a REDcap survey.^21–27^ This present study examined caregiver responses to questionnaires regarding sociodemographics, birth history, cCMV testing and present medical, behavioral, and developmental characteristics (available from the authors upon request). All data were gathered by caregiver report. There is evidence of reliability and validity of parent recollection of pregnancy and birth complications,^28^ and consistency over time.^29^ The literature also supports the reliability of parent-reported developmental and behavioral diagnoses, as compared to the medical record.^30–32^

### Sociodemographic characteristics

Characteristics collected included caregiver age, sex, relationship to the child, and geographic location, as well as the child’s age and sex. In terms of birth history, participants reported their recollection of the child’s symptoms of cCMV at birth (present vs. absent), whether the child required a stay in the neonatal intensive care unit, and whether the child received antiviral treatment in infancy. Caregivers also reported the timing of their child’s testing for cCMV and recalled reasons that prompted cCMV testing.

### Disease severity

We created a variable based on the reported signs and symptoms at birth, called Severity Status, which was based on consensus statement criteria.^33^ Children with a Severity Status of Symptomatic/cCMV Disease were reported to have one or more of the following signs at birth based on caregiver response: small for gestational age (SGA) status, microcephaly, petechiae, hepatomegaly, splenomegaly, abnormal brain imaging (e.g., calcifications, polymicrogyria, ventriculomegaly, cysts), chorioretinitis and/or seizures. Prematurity alone was not classified as Symptomatic. Children with a Severity Status of Asymptomatic/cCMV Infection with Isolated SNHL had no signs at birth, but did refer on their newborn hearing screen (NBHS) and have SNHL identified before 1 year of age. Those with Severity Status of Asymptomatic/cCMV Infection had no signs, abnormal imaging studies or SNHL detected at <1 year.

### Child medical, developmental, and behavioral status

Lastly, caregivers reported their child’s present cCMV-related medical sequelae (present vs. not present), behavioral and developmental diagnoses.

### Analysis

We performed statistical analyses using SAS 9.4 (SAS Institute, Cary, NC). We used latent class analysis (LCA) to determine empirical groups of neurodevelopmental and medical outcomes. Latent class models are used to identify a relatively small number of underlying classes in which to group individuals according to a larger number of observed variables. The resulting latent classes can then be tested and described by their ability to separate individuals by their covariates, or neurodevelopmental and medical outcomes as in this study. In the present study, we used binary responses to 26 different neurodevelopmental and medical diagnoses to determine the latent classes of ND outcomes, which we called ND Phenotypes. We decided on the number of latent classes by balancing model fit (using the Bayesian Information Criterion) and the size of latent classes (Table 1).

**Table 1:** Fit indices for the ND Phenotype analysis of neurodevelopmental and medical outcomes of children with congenital cytomegalovirus.

Number of classes	AIC	BIC	aBIC	Entropy
1	4042.53	4122.22	4033.46	1.00
2	3050.96	3249.83	3069.15	0.94
3	2923.05	3223.10	2950.49	0.87
4	2844.37	3245.59	2881.06	0.90
5	2770.48	3272.89	2816.43	0.93

Statistical Measures to Compare the fit of different models with different numbers of classes:

*AIC* Akaike Information Criterion, *BIC* Bayesian Information Criterion, *aBIC* Adjusted Bayesian Information Criterion^55^.

*Entropy* measures how well the latent classes separate from each other, a higher entropy indicates better class distinction^56^.

*Interpretation*: in general when comparing models with different numbers of latent classes, the model with the lowest AIC, BIC or aBIC is considered the best choice based on the data.^55^ Entropy must also be taken into consideration with fit indices when selecting a final model^56^.

Lastly, we assessed whether latent class membership could be predicted by a child’s sociodemographic characteristics, birth characteristics or reasons for cCMV testing using chi-square analyses.

## Results

### Sociodemographic characteristics

The sociodemographic characteristics of the cohort are presented in Table 2. Of the 242 caregivers, most were female (98.8%), identified as being the mother of the child with cCMV (93.4%), and were from North America (79.8%). Mean caretaker age was 36 years old (SD 7.4 years). Of the children, about half were female (52.5%) and were reported to be between the ages of 4 and 10 years (55.8%).

**Table 2:** Sociodemographic and birth characteristics (*N *= 242).

Sociodemographic characteristics	*N*	%
*Child age (years)*		
1– 3	107	44.2
4–10	135	55.8
*Child is female (vs. male)*	127	52.50
*Caregiver is female (vs. male)*	239	98.8
*Caregiver age (years); mean SD*	36.0	7.4
*Caregiver is mother (vs. other relationship)*	226	93.4
*Geographic location*		
Australia	10	4.1
Europe	33	13.6
North America	193	79.8
Other	6	2.5%
*Birth, pregnancy and cCMV-related characteristics*		
*During pregnancy, cCMV was:*		
Not diagnosed or suspected	167	69.0
Yes, confirmed	38	15.7
Yes, suspected	36	14.9
Unsure	1	0.4
*Mother received antivirals during pregnancy*^a^	15	20.3
*Infant born preterm*	52	21.50
*Admitted to the NICU*	100	41.3
*Received antiviral medication in infancy (vs. not)*	161	66.5
*Disease Severity*^b^		
Symptomatic/cCMV Disease	179	73.97
Asymptomatic/cCMV Infection	32	12.22
Asymptomatic/cCMV Infection with Isolated SNHL	31	12.81
*Signs at birth (present vs. not)*		
Enlarged liver and/or spleen	49	20.2
Thrombocytopenia	78	32.2
Abnormal brain imaging (calcifications, polymicrogyria, ventriculomegaly, cysts etc.)	118	48.8
Petechiae	87	36.0
Jaundice	73	30.2
Microcephaly	82	33.9
Elevated liver enzymes	45	18.6
Seizures	14	5.8
Low birth weight/SGA	95	39.3
Hypotonia or hypertonia	55	22.7
Poor sucking/feeding problems	60	24.8
Referred newborn hearing screen	152	62.8
None	16	6.6
*Timing of cCMV diagnosis*		
Newborn period	179	74.0
Later in infancy or childhood (>60 days)	63	26.0
*Reason for cCMV testing (select all that apply):*		
Known maternal CMV exposure	36	14.9
Abnormalities seen on fetal ultrasound	66	27.3
Referred hearing screening	94	38.8
Preterm	38	15.7
Admitted to the NICU	59	24.4
Testing was routine at birth hospital	10	4.1
Participated in CMV screening study	6	2.5
CCMV findings on physical exam	104	43.0
Abnormal test results (labs or imaging)	35	14.5
Developmental delays	32	13.2
Late onset hearing loss	22	9.1

*SD* standard deviation, *cCMV* congenital CMV, *NICU* neonatal intensive care unit, *SGA* small for gestational age.

^a^*N* = 74, question only asked of mothers with a prenatal diagnosis of cCMV

^b^Severity Status of Definitions:

• Symptomatic/cCMV Disease: ≥1 sign(s) at birth: SGA, microcephaly, abnormal brain imaging, chorioretinitis and/or seizures.

• Asymptomatic/cCMV Infection atic had no signs, abnormal imaging studies or SNHL detected at <1 year.

• Asymptomatic/cCMV Infection with Isolated SNHL had no signs at birth, but did refer on their newborn hearing screen (NBHS) and have SNHL identified before 1 year of age.

### Birth characteristics and reasons for cCMV testing

Birth characteristics are presented in Table 2. Most children were reported to have had a newborn or prenatal diagnosis of cCMV (74.0%). Twenty percent of mothers with a prenatal diagnosis of cCMV received antiviral medication during pregnancy, 21.5% of infants were born preterm (<37 weeks gestational age) and 41.3% required a stay in the neonatal intensive care unit (NICU). At birth, most infants had a Severity Status of Symptomatic/cCMV Disease (*n* = 179, 73.97%), with almost half (*n* = 118, 48.8%) having brain abnormalities on imaging, 39.3% (*n* = 95) were small for gestational age, and over a third (*n* = 87, 36.0%) had petechiae. Over half (*n* = 152, 62.8%) were reported to refer on their NBHS.

### Latent class analysis

**Fig. 1:**
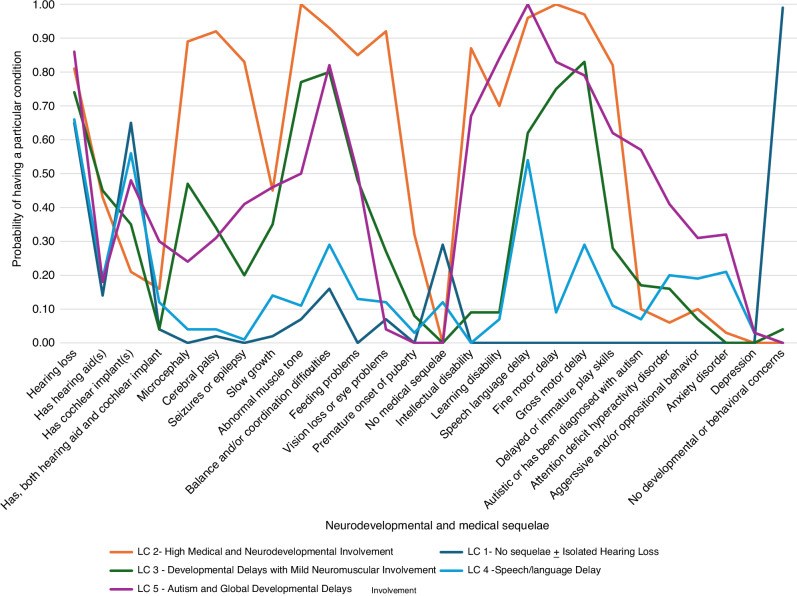
Neurodevelopmental Phenotypes based on medical and neurodevelopmental outcomes in children with congenital cytomegalovirus infection.

The five ND Phenotypes are shown in Table 3 and Fig. 1 showing the probability of each response in each latent class. The ND Phenotypes are described below with the statistically significant associations with sociodemographic and birth characteristics, as well as reasons for cCMV testing (Table 4). Complete Chi-square results testing the association between all characteristics and ND Phenotype membership are shown in the Supplementary Materials (Supplemental Table S1).

**Table 3:** ND phenotype response analysis across levels of neurodevelopmental variables children with congenital cytomegalovirus (*N* = 242).

	Probability of outcome by latent class group					SD Rho^a^	Entire cohort *N* = 242 (%)
	1	2	3	4	5		
	No sequelae ± Isolated SNHL *n* = 44	High Medical and ND Involvement *n* = 31	Developmental Delays with Mild Neuromuscular Involvement *n* = 49	Speech/ language delay *n* = 89	Autism and global developmental delays *n* = 29		
Class size	0.18	0.13	0.20	0.37	0.12	-	1.0
Indicators (present vs. not)							
*Medical sequelae*							
SNHL	0.66	0.81	0.74	0.68	0.86	**0.08**	176 (0.73)
Has hearing aid(s)	0.14	0.43	0.45	0.19	0.18	0.15	47 (0.27)
Has cochlear implant(s)	0.65	0.21	0.35	0.56	0.48	**0.18**	83 (0.47)
Has both hearing aid and CI	0.04	0.16	0.04	0.12	0.30	**0.11**	21 (0.12)
Microcephaly	0.00	0.89	0.48	0.04	0.24	**0.37**	59 (0.24)
Cerebral Palsy	0.02	0.92	0.34	0.04	0.31	**0.36**	49 (0.20)
Seizures or Epilepsy	0.00	0.83	0.20	0.01	0.41	**0.34**	59 (0.24)
Slow growth	0.02	0.45	0.35	0.15	0.46	0.19	97 (0.40)
Abnormal muscle tone	0.07	1.00	0.77	0.11	0.51	**0.41**	126 (0.52)
Balance/Coordination Difficulties	0.16	0.93	0.80	0.30	0.82	**0.35**	77 (0.32)
Feeding Problems	0.00	0.85	0.49	0.13	0.50	**0.34**	57 (0.24)
Vision loss or Eye problems	0.07	0.92	0.27	0.12	0.04	**0.37**	17 (0.07)
Premature onset of puberty	0.00	0.32	0.08	0.03	0.00	0.13	42 (0.17)
No medical sequelae	0.30	0.00	0.00	0.12	0.00	0.13	24 (0.10)
*Developmental, Behavioral and Psychological Sequelae*							
Intellectual Disability	0.00	0.87	0.09	0.00	0.67	**0.41**	51 (0.21)
Learning disability	0.00	0.70	0.09	0.07	0.85	**0.40**	57 (0.24)
Speech Language delay	0.00	0.96	0.61	0.56	1.00	**0.40**	139 (0.57)
Fine Motor Delay	0.00	1.00	0.75	0.10	0.83	**0.45**	101 (0.42)
Gross motor delay	0.00	0.97	0.83	0.30	0.79	**0.41**	121 (0.50)
Delayed or Immature Play skills	0.00	0.82	0.28	0.12	0.62	**0.35**	68 (0.28)
Autism	0.00	0.10	0.17	0.08	0.58	0.23	35 (0.15)
ADHD	0.00	0.06	0.16	0.21	0.41	0.16	40 (0.17)
Aggressive/ Oppositional Behavior	0.00	0.10	0.07	0.20	0.31	0.12	33 (0.14)
Anxiety	0.00	0.03	0.00	0.21	0.32	0.14	29 (0.12)
Depression	0.00	0.00	0.00	0.03	0.03	0.02	4 (0.02)
No developmental or behavioral concerns	0.99	0.00	0.04	0.00	0.00	**0.44**	46 (0.19)

Bold values indicate the SD Rho values that are the most significant contributors to the differences between groups.

^a^SD Rho: a larger value indicates that the variable was more important in sorting participants into latent classes., where 1 indicates a perfect positive correlation, and 0 indicates no correlation.

**Table 4:** Association of statistically significant birth characteristics and reasons that prompted cCMV testing with neurodevelopmental (ND)Phenotype membership in a cohort of children with congenital cytomegalovirus infection^a^.

	1	2	3	4	5	*p* value
	No sequelae ± Isolated SNHL *n* = 44 (18%)	High Medical and Neurodevelopmental involvement 31 (13%)	Developmental delays with mild neuromuscular involvement 49 (20%)	Speech/language delay 89 (37%)	Autism and global developmental delays 29 (12%)	
	*n* (%)	*n* (%)	*n* (%)	*n* (%)	*n* (%)	
*Birth characteristics (present vs. not)*						
Infant born preterm	2 (4.55)	10 (32.26)	18 (36.73)	14 (15.73)	8 (27.59)	0.0007
Admitted to the NICU	8 (18.18)	19 (61.29)	27 (54.10)	27 (30.34)	19 (65.52)	<0.0001
Disease Severity						
Asymptomatic/ cCMV infection	16 (38.10)	1 (3.23)	4 (8.16)	28 (31.82)	1 (3.57)	
Symptomatic/cCMV Disease	26 (61.90)	30 (96.77)	45 (91.84)	60 (68.18)	27 (96.43)	<0.0001
Thrombocytopenia	5 (11.36)	16 (51.61)	19 (38.78)	24 (26.97)	14 (48.28)	0.0004
Abnormal brain imaging^b^	10 (22.73)	26 (83.87)	27 (55.10)	36 (40.45)	19 (65.52)	<0.0001
Petechiae	12 (27.27)	21 (67.74)	21 (42.86)	18 (20.22)	15 (51.72)	<0.0001
Microcephaly	4 (9.09)	27 (87.10)	26 (53.06	12 (13.48)	13 (44.83)	<0.0001
Elevated liver enzymes	3 (6.82)	11 (35.48)	9 (18.37)	17 (19.10)	5 (17.24)	0.04
Seizures	1 (2.27)	6 (19.35)	3 (6.12)	2 (2.25)	2 (6.90)	0.01
Low birth weight/SGA	4 (9.09)	18 (58.06)	28 (57.14)	29 (32.58)	16 (55.17)	<0.0001
Hypotonia or hypertonia	4 (9.09)	18 (58.06)	15 (30.61)	14 (15.73)	4 (13.79)	<0.0001
*Reason for cCMV testing*						
Abnormalities seen on fetal ultrasound	5 (11.36)	13 (41.94)	11 (22.45)	18 (20.22)	19 (65.52)	<0.0001
*Preterm*	0 (0.00)	8 (25.81)	13 (26.53)	9 (10.11)	8 (27.59)	<0.0001
Admitted to the NICU	3 (6.82)	15 (48.39)	16 (32.65)	16 (17.98))	9 (31.03)	0.0002
cCMV findings on physical exam	9 (20.45)	23 (74.19)	24 (48.98)	27 (30.34)	21 (72.41)	<0.0001
Abnormal test results (labs or imaging)	3 (6.82)	8 (25.81)	6 (12.24)	8 (8.99)	10 (34.48)	0.0033

*NICU* neonatal intensive care unit, *SGA* small for gestational age, *cCMV* congenital cytomegalovirus.

^a^Variables not found to be of a statistically significant difference between the groups are included in the full table in the Supplementary Materials.

^b^Defined as “calcifications, polymicrogyria, ventriculomegaly, cysts etc.” in the survey.

There were no differences between any ND Phenotypes based on child sex, child age, caregiver sex, caregiver age or geographic location.

### ND phenotype 1—no sequelae with or without Isolated SNHL (*n* = 44, 18%)

Like all classes, children belonging to ND Phenotype 1 had a probability of SNHL (0.65). In general, children in this ND Phenotype had a higher probability of having no medical sequelae of cCMV (0.29) relative to the other ND Phenotypes. Children in this group had close to zero probability of having a reported neurodevelopmental, behavioral, or psychiatric concern (0.01 vs. 0.96–1.00).

Children in this ND Phenotype were the least likely to have been born prematurely (5%) or be admitted to the NICU (18%) relative to other ND Phenotypes (*p* < 0.0001, Table 4). However, only 11% of caregivers of children in this ND phenotype reported that their child had no signs or symptoms of cCMV at birth (Table 4). In terms of reasons for testing, most children in ND Phenotype 1 were tested for cCMV due to a referred NBHS (43%) or known maternal exposure to CMV in pregnancy (18%), although there were no statistically significant differences between ND Phenotypes. Children in this ND phenotype were the least likely to have been tested for cCMV due to findings on physical exam at birth relative to other ND Phenotypes (20% vs. 30–74%, *p* < 0.0001).

### ND phenotype 2—high medical and neurodevelopmental involvement (*n* = 31, 13%)

Children belonging to ND Phenotype 2 had a high probability of medical sequelae stemming from central nervous system (CNS) involvement including microcephaly (0.89 vs 0.00–0.47), cerebral palsy (0.92, vs 0.02–0.34), and seizures (0.83 vs. 0.00 –0.41). All children had abnormal muscle tone (1.0 vs. 0.07–0.77) and many with reported feeding problems (0.85 vs. 0.00–0.50). Children in this ND Phenotype had a high probability of SNHL (0.81) and vision problems (0.92 vs 0.04–0.24), meaning that many (77%) were likely Deafblind to a degree. All children in ND Phenotype 2 had some neurodevelopmental or behavioral concerns. The probability of having an intellectual disability in this ND Phenotype was 0.87 (vs. 0.00–0.67). All children in this ND Phenotype had Speech/Language, Gross Motor, and/or Fine Motor delay (0.96–1.00).

Many children in ND Phenotype 2 were born preterm (32%), and/or admitted to the NICU (61%). Almost all (97%) had a Severity Status of Symptomatic/cCMV Disease, had abnormal brain imaging (84%) and/or microcephaly at birth (87%), which where statistically significantly greater than the prevalence of those conditions in the other ND Phenotypes (both *p*’s < 0.0001). Many were reported to have low birth weight for gestational age (58%). The prevalence of several signs of severe symptomatic cCMV/disease was higher in the Phenotype relative to other ND Phenotypes, including abnormal tone (58% vs. 9–32%, *p* < 0.001), seizures (19% vs. 2–7%, *p* = 0.003) and/or elevated liver enzymes (35% vs. 7–19%, *p* = 0.04) at birth. In terms of reasons for testing, many (42%) had abnormalities on fetal ultrasound which prompted testing, preterm birth (26%), admission to the NICU (48%) and/or cCMV findings on physical exam (74%).

### ND phenotype 3—some developmental delays with mild neuromuscular involvement (*n* = 49, 20%)

Those in ND Phenotype 3 had a high probability (0.74) of having SNHL, as well as high rates of abnormal muscle tone (but not cerebral palsy) (0.77) and balance/coordination difficulties (0.80). Children in this group had a 100% probability of having medical sequelae. These children also had elevated probabilities of fine motor, gross motor, and speech/language delays (0.75, 0.83, and. 0.61 respectively). Children in this ND Phenotype had a low probability of having no neurodevelopmental or behavioral sequelae (0.04).

Many children in ND Phenotype 3were born preterm (36%), admitted to the NICU (54%), had thrombocytopenia (38%), petechiae (42%), microcephaly (52%), were low birth weight/small for gestational age (SGA) (32%). Most (96%) had a Severity Status of Symptomatic/cCMV Disease.

### ND phenotype 4—speech and language delays (*n* = 89, 37%)

Children belonging to ND Phenotype 4 had a high probability of SNHL (0.68), but low probabilities of other medical sequelae such as microcephaly (0.04 vs 0.00–0.89), seizures (0.01 vs 0.00–0.83), or vision loss/eye problems (0.12 vs 0.07–0.93), similar to those in ND Phenotype 1. However, unlike ND Phenotype 1, those in ND Phenotype 4 had higher probabilities of having of delays in speech and language (0.56 vs 0.00), gross motor skills (0.30 vs. 0.00). There were no children in ND Phenotype 4 without any developmental or behavioral sequelae, whereas almost all children in ND Phenotype 1 were without developmental or behavioral sequelae (0.00 vs. 0.99).

Relative to the other ND phenotypes, fewer children in ND Phenotype 4 were born preterm (16% vs. 5–36%, *p* < 0.0001) or admitted to the NICU (31% vs. 18–66%, *p *< 0.0001). Roughly a third (32%) had a Severity of Asymptomatic/cCMV infectionat birth. At birth, many infants had abnormal brain imaging (40%), some had microcephaly (13%), or seizures (1%). Reasons for testing for cCMV included abnormalities on fetal ultrasound (20%), referred NBHS (31%) and cCMV findings on physical exam (30%).

### ND phenotype 5—autism and global developmental delay (*n* = 29, 12%)

Finally, children in ND Phenotype 5 had the highest probability of having SNHL (0.86 vs 0.65–0.81) among the other ND Phenotypes. Children in this group had a high probability of having balance/coordination difficulties (0.82 vs. 0.16–0.93), not fully accounted for by the probability of cerebral palsy (0.31 vs 0.00–0.92). There was a high probability of feeding problems relative to the other ND Phenotypes (0.50 vs 0.00–0.85). Children in ND Phenotype 5 had a high probability of having been diagnosed with autism (0.57 vs 0.00–0.17), an intellectual (0.67 vs 0.00–0.87) or learning disability (0.84 vs 0.00–0.70) relative to the other ND Phenotypes. All children had speech/language delays, and most had delays in fine and gross motor skills as well.

In terms of birth characteristics, children in ND Phenotype 5 were more likely to be admitted to the NICU at birth (66% vs. 0–61%, *p* < 0.001), have a Disease Severity of Symptomatic/cCMV Disease (96% vs 52–97%, *p* < 0.001), have abnormal brain imaging (66%), petechiae (52%), and have microcephaly (45%). Most were tested for cCMV due to abnormalities seen on prenatal ultrasound (66%) or having cCMV findings on physical exam (72%).

## Discussion

This study identified five ND Phenotypes in a cohort of children with cCMV: 1) No Sequelae with or without Isolated SNHL, 2) High Medical and Neurodevelopmental Involvement, 3) Some Developmental Delays with Mild Neuromuscular Involvement, 4) Isolated Speech and Language Delays, 5) and Autism and Global Developmental Delay. Childbirth characteristics and recalled reason for cCMV testing were found to be associated with ND Phenotype membership. This is the first study, to our knowledge, which has used Latent Class Analysis to identify distinct phenotypes of ND outcomes within an international cohort of children with cCMV, which allowed for the examination of individual clusters of medical and developmental outcomes, rather than focusing on group trends. This study describes a nuanced spectrum of ND outcome phenotypes, beyond a linear categorization of severity (e.g., mild, moderate, severe). While this cohort is non-representative, with high rates of symptomatic cCMV disease (79%) and brain imaging abnormalities at birth (48%), differences between the ND outcomes in the phenotypes were still broad. These findings highlight the diversity of presentations and outcomes of children with cCMV.

Two phenotypes (ND Phenotype 1 No Sequelae with or without Isolated SNHL and ND Phenotype 4 Isolated Speech and Language Delays) had low rates of medical sequelae outside of SNHL, while the other three groups had high levels of medical involvement and developmental delays. Phenotypes with lower levels of medical and developmental sequelae (ND Phenotype 1 and ND Phenotype 4) had relatively lower rates of being born preterm, being admitted to the NICU, and having abnormal head imaging findings at birth as compared to the other phenotypes. These two phenotypes also had the highest rates of Asymptomatic/cCMV Infection severity at birth (38% and 41%) of all the phenotypes. In terms of ND outcomes, the biggest difference between these two groups was the higher rates of speech and language delays in ND Phenotype 4 vs. ND Phenotype 1, even though both groups had similar rates of SNHL (65% vs. 66%). Between these two phenotypes, those in the Isolated Speech Language Delays ND Phenotype had higher rates of birth characteristics that are known risk factors for developmental delays including Symptomatic/cCMV Disease Severity Status, brain imaging abnormalities, being born preterm and spending time in the NICU. Prior studies examining speech and language outcomes in children with cCMV have found high rates of speech and language delays in children with symptomatic cCMV,^3,34,35^ and lower rates of cochlear implant “success” in terms of attaining fluency in oral/aural communication as compared to children with genetic SNHL.^36–38^

ND Phenotypes 2, 3 and 5 (High Medical and Neurodevelopmental Involvement, Some Developmental Delays with Mild Neuromuscular Involvement, and Autism and Global Developmental Delay, respectively) had similar rates of being born preterm, being admitted to the NICU, and almost all children had some signs or symptoms reported at birth. Like other studies that have found microcephaly and abnormal brain imaging at birth to be associated with greater developmental delays, we found children in ND Phenotype 2 (High Medical and Neurodevelopmental Involvement) had relatively higher rates of microcephaly and abnormal brain imaging reported at birth compared to other ND phenotypes. While overall there were relatively high rates of abnormal brain imaging reported in ND Phenotypes 2, 3 and 5 (84%, 54%, and 66% respectively), children in ND Phenotype 2 had higher rates of features at birth that may suggest more severe brain involvement (seizures, abnormal tone, microcephaly). Of note, children in ND Phenotype 2 had high rates of combined SNHL and vision loss, which depending on the severity, can have significant implications on well-being, communication and learning.^39^ This study did not examine the extent of CNS involvement in our participants, however, other research has found the degree of CNS involvement to be associated with ND outcomes.^40,41^ It may be that severity of CNS involvement is a key factor in differentiating ND Phenotypes 2, 3 and 5.

Over half of the children in ND Phenotype 5 had a reported diagnosis of autism. This ND Phenotype had the highest rates of referring on the NBHS and having SNHL. In the greater cohort, 14% of children were reported by parents to have autism. Recent work from our group using administrative claims data found a 2.5-fold increased risk of autism in children with cCMV, that was not fully explained by CNS involvement, preterm birth or low birth weight.^42^ The mechanism by which cCMV may increase the risk of autism is unknown, although some have speculated about the virus’s impact on the developing fetal brain along with epigenetic factors.^43–45^ Children with SNHL in general have been found to have higher rates of autism.^46^ It remains unknown whether early SNHL and resultant reduced access to language during critical developmental periods may compound the risk of autism in children with other risk factors, such as epigenetics and congenital infections.^47,48^ All children in ND Phenotype 5 had language delays, which is unsurprising given their compounding risk factors of autism and SNHL. Language acquisition in deaf-autistic children has been a focus of recent research, as these children often require specific and unique accommodations.^49–54^ Future studies should examine timing of autism and SNHL diagnoses as well as response to therapeutic supports on functional outcomes in children with cCMV.

While results of this study cannot be generalized to all children with cCMV, clinicians may consider monitoring for additional sequelae of cCMV especially as some concomitant conditions may be challenging to diagnose. Future research regarding the stability of ND Phenotypes, and the role of early intervention as well as whether more nuanced birth characteristics and biomarkers can predict later ND Phenotype is necessary to bring the science closer to being able to provide specific anticipatory guidance for parents of infants with cCMV.

Strengths of this study include a large international cohort of caregivers of children with cCMV. The cohort, however, was not a representative sample, with a high number of children who were reported to haveSymptomatic/cCMV Disease at birth and with long-term sequelae. Furthermore, as universal screening is still not common in the United States, most infants with cCMV infection are likely to go undetected at birth and for the rest of their lives, as most will not develop sequelae to prompt later testing. Caution should be taken in interpretation of study findings, which may be best applied to those with Symptomatic cCMV disease and hearing differences. Furthermore, like most studies of children with cCMV, this study did not measure many factors that are known to be important in developmental outcome such as maternal education, access to early intervention, outpatient therapies, complications to NICU course. Lastly, these data are subject to social desirability bias and recall bias, as signs and symptoms, including those that may not be as salient to the general population, were based on caregiver report.

## Conclusions

Five ND Phenotypes were found using a child-centered approach in a large cohort comprised mainly of children with Symptomatic Ccmv disease. Birth characteristics suggestive of more severe CNS involvement (e.g., seizures, microcephaly) were associated with the High Medical and Neurodevelopmental Involvement Phenotype. Despite a high rate of CNS anomalies and symptoms at birth in this cohort, almost half of children belong to ND Phenotypes with no sequelae, isolated SNHL or isolated speech/language delay. Future studies should examine whether ND Phenotypes are stable, and the impact of early interventions on ND Phenotypes.

## Supplementary information

Supplementary Table S1

## Data Availability

Deidentified data is available from the authors upon request and with appropriate Institutional Review Board approval.
